# Identification of the *Yellow Skin* Gene Reveals a Hybrid Origin of the Domestic Chicken

**DOI:** 10.1371/journal.pgen.1000010

**Published:** 2008-02-29

**Authors:** Jonas Eriksson, Greger Larson, Ulrika Gunnarsson, Bertrand Bed'hom, Michele Tixier-Boichard, Lina Strömstedt, Dominic Wright, Annemieke Jungerius, Addie Vereijken, Ettore Randi, Per Jensen, Leif Andersson

**Affiliations:** 1 Department of Medical Biochemistry and Microbiology, Uppsala University, Uppsala, Sweden; 2 INRA, AgroParisTech, UMR1236 Génétique et Diversité Animales, Jouy-en-Josas, France; 3 Department of Animal Breeding and Genetics, Swedish University of Agricultural Sciences, Uppsala, Sweden; 4 Hendrix Genetics, Breeding Research & Technology Centre, Boxmeer, The Netherlands; 5 Istituto Nazionale per la Fauna Selvatica, Laboratorio di Genetica, Ozzano Emilia, Italy; 6 IFM Biology, Linköping University, SE-58183 Linköping, Sweden; University of Liège, Belgium

## Abstract

Yellow skin is an abundant phenotype among domestic chickens and is caused by a recessive allele (W*Y) that allows deposition of yellow carotenoids in the skin. Here we show that yellow skin is caused by one or more cis-acting and tissue-specific regulatory mutation(s) that inhibit expression of *BCDO2* (beta-carotene dioxygenase 2) in skin. Our data imply that carotenoids are taken up from the circulation in both genotypes but are degraded by BCDO2 in skin from animals carrying the *white skin* allele (W*W). Surprisingly, our results demonstrate that *yellow skin* does not originate from the red junglefowl (*Gallus gallus*), the presumed sole wild ancestor of the domestic chicken, but most likely from the closely related grey junglefowl (*Gallus sonneratii*). This is the first conclusive evidence for a hybrid origin of the domestic chicken, and it has important implications for our views of the domestication process.

## Author Summary

Many bird species possess yellow skin and legs whereas other species have white or black skin color. Yellow or white skin is due to the presence or absence of carotenoids. The genetic basis underlying this diversity is unknown. Domestic chickens with yellow skin are homozygous for a recessive allele, and white skinned chickens carry the dominant allele. As a result, chickens represent an ideal model for analyzing genetic mechanism responsible for skin color variation. In this study we demonstrate that yellow skin is caused by regulatory mutation(s) that inhibit expression of the beta-carotene dioxygenase 2 (BCDO2) enzyme in skin, but not in other tissues. Because BCDO2 cleaves colorful carotenoids into colorless apocarotenoids, a reduction in expression of this gene produces yellow skin. This study also provides the first conclusive evidence of a hybrid origin of the domestic chicken. It has been generally assumed that the red junglefowl is the sole ancestor of the domestic chicken. A phylogenetic analysis, however, demonstrates that though the *white skin* allele originates from the red junglefowl, the *yellow skin* allele originates from a different species, most likely the grey junglefowl. This result significantly advances our understanding of chicken domestication.

## Introduction

**Figure 1:**
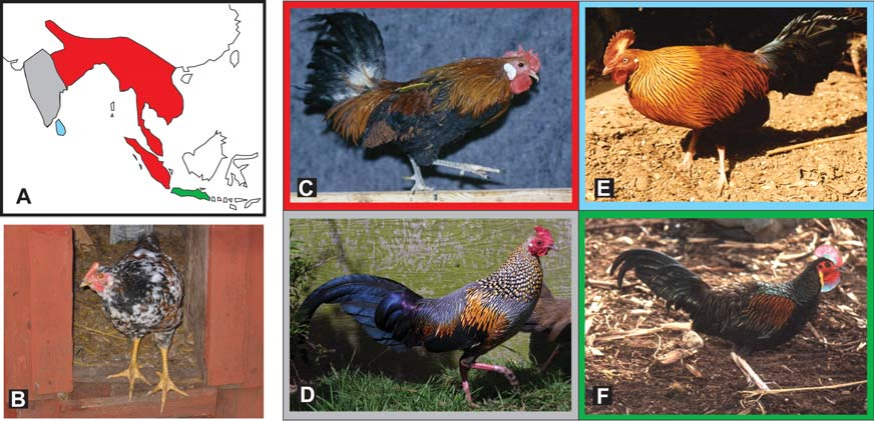
Adapted from [2],[17]. Panel A depicts a map of South Asia onto which the ranges of four species of junglefowl are drawn. Panel B depicts a European domestic chicken with yellow legs. Red, grey, blue, and green regions represent the respective ranges of red, grey, Ceylon, and green junglefowls. Images of these birds are presented in panels C through F respectively, within colored borders that correspond to the colors on the map. (Photo: Figures 1B: Björn Jacobsson; 1C: Erik Bongcam-Rudloff; 1D: John Corder, World Pheasant Association; 1E: Jean Howman, World Pheasant Association; 1F: Kenneth W Fink, World Pheasant Association).

The origin of the domestic chicken has been under debate for centuries [1]. Not only has the geographical center of the first (and possible additional) domestication event remained contentious [1]–[3], but because several closely related species of junglefowls exist in South Asia (Figure 1), the possibility that chickens originate from multiple wild ancestors has yet to be eliminated. On the basis of observed character differences and cross-breeding experiments, Darwin concluded that domestic chickens were derived solely from the red junglefowl [4], though this was later challenged by Hutt [1], who stated that as many as four different species of junglefowls may have contributed to chicken domestication. Molecular studies of mtDNA [5] and retroviral insertions [6] have supported Darwin's view. A study that analyzed both repeat nuclear elements and mitochondrial sequences found evidence that grey and Ceylon junglefowls may hybridize with domestic chickens, but did not provide evidence that these two species have contributed to chicken domestication [7]. To date, no studies have compared gene sequences associated with a specific phenotype found in domestic chickens across numerous wild junglefowls and domestic breeds.

The majority of chickens used for commercial egg and meat production in the Western world are homozygous for the *yellow skin* allele. In live birds, the phenotype is most easily recognized by the presence of yellow legs. The expression of yellow skin is influenced by the amount of carotenoids, primarily xanthophylls, in the feed [1]. More carotenoids produce a more intense yellow color. There is a strong consumer preference for the yellow skin phenotype in certain geographic markets such as USA, Mexico, and China where synthetic pigment may be added to enhance the yellow color [8],[9]. Carotenoids also play a crucial role for feather or skin pigmentation in some wild birds, a well-known example of which is the flamingo's pink feathers. Carotenoid-based ornaments (skin or feathers) in wild birds are considered to be an honest signal of an individual's nutritional status or health, reflecting its foraging efficiency or immune status and are therefore implied to affect sexual attractiveness [10]–[12]. A better understanding of the molecular mechanisms regulating the distribution of carotenoid pigmentation is therefore of considerable interest for evolutionary genetics.

## Results

### Positional Identification of *Yellow Skin*

**Figure 2:**
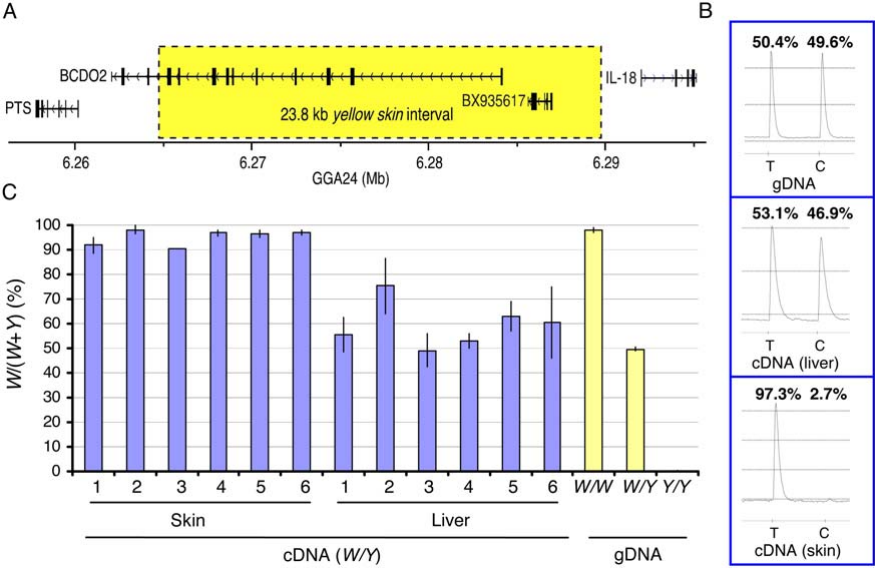
(A) Gene content of the *yellow skin* interval. The 23.8 kb region showing complete association with *yellow skin* is indicated by a box. The annotation is based on the chicken genome assembly as presented on the UCSC server (http://genome.ucsc.edu; Build May2006). (B) Differential expression of the *BCDO2* transcript in skin but not liver from *yellow skin* heterozygotes using genomic DNA (gDNA) as control. The polymorphic position chr24:6,268,434 bp was used to monitor differential expression using pyrosequencing. T and C at this position correspond to the *white* and *yellow skin* alleles respectively. (C) Summary of the examination of differential expression in skin and liver from six heterozygous (*W/Y*) birds. Genomic DNA from the three different genotypes were used as controls.

The gene underlying *yellow skin* was identified by combining linkage analysis and Identical-by-Descent (IBD) mapping across breeds with the yellow skin phenotype; IBD mapping was carried out with the assumption that the *yellow skin* mutation has for most breeds, if not all, been inherited from a common ancestor. *yellow skin* was previously assigned to chromosome 24 [13]. A *Y/W*×*Y/Y* back-cross pedigree, comprising 91 informative meioses, was used to refine the map position of the locus. Close linkage was detected to a single nucleotide polymorphism (SNP) located within *APOA1* at nucleotide position 5,237,523 bp at the distal end of chromosome 24 (lod score = 16.4; recombination fraction = 6.9%). An examination of this chromosomal region revealed an obvious candidate gene for yellow skin, *BCDO2* located at position 6.26–6.29 Mbp. *BCDO2* encodes beta-carotene dioxygenase 2, an enzyme that cleaves colorful carotenoids to colorless apocarotenoids by an asymmetric cleavage reaction [14]. Partial sequence analysis of *BCDO2* immediately revealed a SNP in complete linkage disequilibrium with *yellow skin* across a divergent set of breeds (Table 1). This highly significant association across breeds and complete fixation within breeds homozygous for *yellow skin* confirmed our assumption that this allele has been inherited from a single ancestor. Thus, the causal mutation should be located within the minimum shared haplotype present in these breeds. Further sequence analysis revealed that this minimum haplotype spans 23.8 kb between nucleotide positions 6,264,083 to 6,287,900. In addition to *BCDO2*, this region only contains one other putative gene corresponding to a single chicken cDNA clone (BX935617; Figure 2A).

**Table 1:** Allele frequencies at SNPs around *BCDO2* on chicken chromosome 24 among birds with the yellow or white skin phenotype.

Breed	n	SNP^1^		
		A	B	C
**Yellow skin**				
White Leghorn, line 13	5	1.00	1.00	1.00
White Leghorn, OS line	5	1.00	1.00	1.00
White-egg layer A^2^	8	0.94	1.00	0.94
Brown egg layer B^2^	7	1.00	1.00	1.00
Brown egg layer D^2^	7	1.00	1.00	1.00
Broiler sire line D^2^	8	0.69	1.00	0.88
Broiler dam line D^2^	7	1.00	1.00	1.00
White Plymouth Rock	6	1.00	1.00	1.00
Godollo Nhx^2^	8	0.81	1.00	0.81
Orlov^2^	12	0.75	1.00	0.71
**White skin**				
Friesian Fowl^2^	6	0	0	0.33
Padova^2^	4	0	0	0.38
Westfälischer Totleger^2^	3	0	0	0
Houdan^2^	5	0	0	0
Dorking^2^	4	0	0	0.13
Red Villafranquina^2,4^	5	0.10	0.10	0.20
Czech Golden Pencilled^2^	5	0	0	0
Australorp^2^	5	0	0	0.10
Red junglefowl^3^	24	0.04	0	0.29

n = number of individuals

1 SNP A = nucleotide position 6,264,085, G/**A**; SNP B = nucleotide position 6,273,428, A/**G**; SNP C = nucleotide position 6,287,900, G/**A**; bold, underlined nucleotides are those associated with the *yellow skin* haplotype. The *BCDO2* gene spans from nucleotide position 6,262,596 to 6,282,641 bp.

2 These samples were collected by the AvianDiv project [28]

3 The red junglefowl data include the genotype deduced from the genome assembly as presented on the UCSC server (http://genome.ucsc.edu; Build May2006)

4 One sample was heterozygous at SNP B and apparently carried the *yellow skin* allele.

### Differential Expression of Alleles in Skin

RT-PCR analysis revealed only weak expression of the transcript corresponding to BX935617 and no significant difference between genotypes was documented (data not shown). *BCDO2* showed fairly strong expression in both liver and skin. RT-PCR analysis followed by pyrosequencing of six heterozygous birds demonstrated that more than 90% of the transcripts expressed in skin originated from the *white skin* allele whereas *yellow skin* and *white skin* was expressed at about the same level in liver (Figures 2B and 2C). We postulate that *yellow skin* is caused by tissue-specific regulatory mutation(s) that alter *BCDO2* expression in skin. Yellow carotenoids are assumed to be taken up to skin in both genotypes but in white skin birds the carotenoids are degraded to colorless apocarotenoids by the action of BCDO2.

### Phylogenetic Analysis

**Figure 3:**
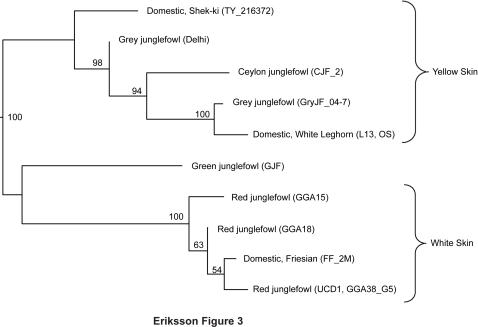
A neighbor-joining tree depicting the relationships between sequences derived from 23.8 kb of the *BCDO2* locus. In total, eleven birds were re-sequenced (the identity number of each bird is given in parenthesis); UCD1 represents the reference genome sequence. Wild and domestic samples possessing white and yellow skin clearly separate into two divergent clades. Node support values were generated from 1000 bootstrap replicates. The relative position of the Grey junglefowl (Delhi) is a result of the fact that this sample was heterozygous for two alleles, one of which most closely matched GryJF_04-7 and another which more closely matched TY_216372. Heterozygous positions were coded using degenerate bases and thus the algorithm used to draw the neighbor-joining tree placed this sample into a relatively basal position. Figure S7 shows the differing nucleotide positions across this region.

We searched for the causal mutation(s) by resequencing the entire 23.8 kb region from domestic chickens homozygous for *yellow skin* together with a set of domestic chickens and red junglefowls homozygous for *white skin*. This analysis revealed a surprisingly high sequence diversity between the two groups (0.81%), well above the genome average for chicken (∼0.5%) [15] and approaching the sequence divergence between chimpanzee and human (1.2%) [16]. We therefore included three other species of junglefowls in the sequence comparison: grey (*G. sonneratii*), Ceylon (*G. lafayetii*), and green (*G. varius*) junglefowls. This step was also motivated by the fact that grey and Ceylon junglefowls have red or yellowish legs which implies deposition of carotenoids and a *Y/Y* genotype [17]. This had previously prompted Hutt [1] to propose that *yellow skin* may have been derived from the grey junglefowl. The *white skin* allele from domestic chicken showed a high sequence identity to red junglefowl sequences whereas the *yellow skin* sequences clearly clustered with sequences from grey and Ceylon junglefowls (Figure 3); *Y* showed only 13 nucleotide differences (0.07%) and three insertions/deletions compared with one of the grey junglefowl sequences.

In contrast, mtDNA sequences from the same samples showed the expected pattern in which domestic chickens cluster with red junglefowl within a clade well separated from other junglefowls (Figure S1). We reanalyzed previously published sequences [7] of chicken repeat 1 elements spread across the genome and demonstrated that trees constructed using three separate regions on chromosome 1, and another on chromosome 5, possessed the same approximate topology as revealed by an analysis of mitochondrial sequences (Figures S2, S3, S4, and S5). On all of these trees, domestic chicken haplotypes cluster exclusively with those of red junglefowl. In contrast, a tree drawn using a region located about ∼650 kb proximal to *BCDO2* gave an inconclusive picture (Figure S6) consistent with our finding that introgression from other junglefowl species has affected this region of chromosome 24.

Partial resequencing of the 23.8 kb region revealed that all Western breeds fixed for *yellow skin* carried the same haplotype. We also resequenced haplotypes present in Chinese Shek-ki birds, all of which express the yellow skin phenotype, and found that they carried a distinct haplotype. This haplotype clustered with sequences from grey and Ceylon junglefowls, and could therefore represent a distinct introgression event (Figure 3). We analyzed five birds from this breed and four were homozygous for the alternate *yellow skin* haplotype whereas the fifth was a composite heterozygote between the alternate and the haplotype found in European breeds demonstrating that yellow skin is controlled by the same locus in Asian and European breeds. The fact that *yellow skin* is present among local breeds of domestic chicken across the world suggests that introgression of *yellow skin* to domestic chickens happened thousands of years ago rather than hundreds of years ago.

We also resequenced ∼3 kb of the *BCDO2* region from six additional grey junglefowls and all sequences belonged to the *yellow skin* cluster; three of the sequences clustered with the grey junglefowl (Delhi) sequence and the other three clustered with grey junglefowl (GryJF_04-07) (data not shown). In conclusion, all eight tested grey junglefowls were homozygous for alleles that were closely related to the *yellow skin* allele. In contrast, our SNP screen showed that all 24 tested red junglefowls carried alleles at this locus that are closely related to *white skin* alleles in domestic chicken (Table 1).

### Quantitative Trait Locus (QTL) Analysis

Billions of chickens used for producing meat (broilers) and eggs (layers) are homozygous *Y/Y*, though it is unclear why *yellow skin* has almost replaced the *white skin* allele in commercial populations. We tested whether *yellow skin* may have pleiotropic effects on other traits using our large intercross between the red junglefowl (*W/W*) and White Leghorn chickens (*Y/Y*) [18]. The pedigree comprises about 800 F_2_ progeny which have been scored for a number of phenotypic traits including growth, body composition, egg production, bone density, and behavior. The results of the QTL analysis using the *BCDO2* marker are compiled in Table S3. We tested a total of 81 traits and only four reached nominal significance, which is not more than expected by chance alone. Thus, no highly significant trait association was detected for the *BCDO2* locus taking into account the number of tests performed. The most interesting association in relation to the selection for *yellow skin* in domestic chickens was the slightly higher egg production in birds carrying this allele. However, the statistical support for this association was weak and requires additional investigations.

## Discussion

This study convincingly demonstrates that while domestic chickens inherited the mitochondrial, and most of their nuclear genome from red junglefowl, the *yellow skin* allele originates from a species of junglefowl other than the red junglefowl, most likely from the grey junglefowl. The alternative explanation that *W* and *Y* haplotypes have been segregating within red junglefowl populations for a sufficient period of time to have accumulated the observed sequence divergence can be ruled out because the *yellow skin* sequence is too similar to the grey junglefowl sequence. As shown in Figures S1, S2, S3, S4, and S5, mtDNA and nuclear sequences from the grey junglefowl are clearly distinct from those found in red junglefowl and domestic chicken. The only exception to this rule detected so far is the *yellow skin* locus. For instance, the divergences between sequences from the grey and red junglefowl are generally similar to the sequence divergence between red and green junglefowl (Figure 1 and Figures S1, S2. S3, S4, S5, and S6). In contrast, the minimal sequence divergence between the grey junglefowl *BCDO2* sequence and the domestic *yellow skin* allele makes it highly unlikely that the divergence between the *white* and *yellow skin* alleles predates the speciation of the red and grey junglefowl; the *Y* sequence would have accumulated numerous sequence differences since the split between the red and grey junglefowl. We cannot exclude the possibility that *yellow skin* was introgressed to the red junglefowl by hybridization with grey junglefowl prior to domestication, but it is much more plausible that introgression was facilitated by human activities. The red and grey junglefowls are full species as demonstrated by the fact that hybridization does not occur in the wild [17] and when attempted in captivity, only a cross between grey cocks and red hens produced mostly sterile offspring [19]. Hybridization between grey junglefowl and domesticated fowl, however, have been reported in the vicinity of villages within the area of contact between the two wild species [17], suggesting that the introgression of yellow skin into domestic birds took place after chickens were initially domesticated.

A QTL analysis did not reveal any convincing QTL effects associated with the segregation at the *yellow skin* locus in an intercross between the red junglefowl and White Leghorn chickens. This result is consistent with a previous back-cross experiment which did not reveal any significant difference in body weight or egg production between *W/Y* and *Y/Y* birds [20]. However, studies in other species have indicated that access to carotenoids is a limiting factor for egg-laying capacity [21]. During lay, carotenoids are mobilized and deposited in the yolk of the egg. It is therefore worth speculating that the bright yellow skin color, expressed by well-fed *yellow skin* homozygotes but not by well-fed *white skin* birds, has been associated with high production and good health at some point during domestication and was therefore favored by early farmers. Of course, *yellow skin* may also have been selected purely for cosmetic reasons.

This study also contributes to the accumulating data supporting King's and Wilson's [22] conjecture of the importance of regulatory mutations as a source for phenotypic variation. Because *BCDO2* is expected to have an essential role for the Vitamin A metabolism in vertebrates [14], loss-of-function mutations may cause severe defects or lethality, whereas a tissue–specific regulatory mutation, like the one presented here, can be tolerated more readily. Other examples of regulatory mutations with important phenotypic effects include a substitution in *IGF2* leading to higher muscle-specific expression in pigs [23] and a *Pitx1* mutation leading to reduction in pelvic size in sticklebacks [24].

The mutation(s) causing the yellow skin phenotype must be located within the 23.8 kb region which shows complete association with the yellow skin phenotype across breeds (Figure 2A). The identification of the mutation(s) for *yellow skin* is hampered by two facts: 1) this phenotype is not caused by a recent mutation event but instead represents a species difference that may involve multiple substitutions with phenotypic effects, and 2) it is not clear whether the red junglefowl allele represents the ancestral or derived state. At present there are 115 fixed nucleotide substitutions between the clusters of *yellow skin* and *white skin* sequences; one of these is a missense mutation (K416N) but it affects a residue that is not well conserved between species. Sequence data from more distantly related bird species like the zebra finch are required to identify evolutionary conserved regulatory elements where the causal mutation may reside. An obvious topic for future research is to study the role of *BCDO2* in carotenoid pigmentation variation in birds, fish, and other vertebrates, including humans. In fact, the presence of yellow fat has been shown to be inherited as a recessive trait in both rabbits [25] and sheep [26]. *BCDO2* is now an obvious candidate gene for these traits.

This study contradicts the assumption that the red junglefowl is the sole wild ancestor of the domestic chicken [5] and provides the first conclusive evidence that other species have contributed to the domestic chicken genome. We therefore propose that the taxonomy of the domestic chicken should be changed from *Gallus gallus domesticus* to *Gallus domesticus* to reflect the polyphyletic origin of chicken [27]. The emerging technologies for total genome resequencing can be readily employed to determine if other parts of the chicken genome also originate from other species of junglefowls. Such regions are expected to be enriched for functionally important variants, like *yellow skin*, because neutral sequences should have been diluted out during the extensive back-crossing that must have taken place after introgression. It is possible that the introgression of *yellow skin* was facilitated by the fact that it resides on a microchromosome (only 6.4 Mb in size) with a high recombination rate, which reduces the amount of genetic material affected by linkage drag.

## Materials and Methods

### Animals

DNA samples from a pedigree comprising 91 informative meiosis from a W*W/W*Y×W*Y/W*Y backcross, collected by Hendrix Genetics B.V. (Holland), were used for the linkage analysis. DNA samples from various domestic breeds collected by the AvianDiv project [28] were used for IBD mapping together with samples from experimental populations used by the Uppsala group. The origin of samples from different species of junglefowl is shown in Table S1 together with information on the domestic chicken included in this study.

Tissues from breast skin and liver used in the expression analysis were sampled from an experimental cross at the INRA experimental station (Tours, France) segregating for *yellow skin*. Tissues were kept in −70°C until the expression analysis was performed.

### SNP Analysis

A single nucleotide polymorphism (SNP) at position chr24:5,237,523 (A→G), was genotyped in 91 individuals from the pedigree material provided by Hendrix Genetics B.V by single base extension; primer sequences are given in Table S2. All other SNP typings were done by pyrosequencing using the Pyro Gold chemistry (Biotage, Uppsala, Sweden) and the PCR and sequencing primers are listed in Table S2.

### Resequencing

The 23.8 kb region was resequenced in eleven chickens. Three of these were expected to be homozygous for *yellow skin* (White Leghorn Line 13 from Uppsala, White Leghorn OS strain, and Chinese She-ki) while four were expected to be homozygous *white skin* (a Friesian Fowl and samples from three different subspecies of red junglefowl, *Gallus gallus gallus, Gallus gallus jabouillei* and *Gallus gallus bankiva*). Furthermore, samples from two grey junglefowls (*Gallus sonneratii*) and one Ceylon junglefowl (*Gallus lafayetii*), both expected to be homozygous for *yellow skin* on the basis of leg color, were included. A sample of green junglefowl (*Gallus varius*) was also included in the sequence comparison though we have no reliable information of the leg color of this species.

All primers pairs used to generate overlapping PCR amplicons ranging between 700–1000 bps in size are shown in Table S2 and they were designed using the Primer3 software [29]. The same primers were also utilized for sequencing. The sequences were analyzed and edited with Codon Code Aligner (CodonCode, Dedham, MA). The sequence from the red junglefowl used to generate the chicken genome sequence was downloaded from GenBank and used as a reference in the alignment.

### mtDNA Analyis

The D-loop of mitochondrial DNA was PCR-amplified and sequenced from a number of domestic and wild chickens. The PCR primers GalCR_L16750 and GalCR_rev [5] were used to amplify a 1325 bp fragment. Sequencing primers are listed in Table S2.

### Expression Analysis

Tissues (skin and liver) were collected from animals being *yellow skin* heterozygotes (confirmed by genotyping of SNP chr24:6,268,434). Total RNA was extracted from skin and liver with TRIzol (Invitrogen, Frederick, MO, USA) and then treated with DNA-*free*™ (Applied Biosystems, Foster City, CA, USA) to remove DNA contamination. The RNA quality was controlled using an Agilent 2100 Bioanalyzer (Agilent Technologies Inc., Santa Clara, CA, USA). The First-Strand cDNA synthesis kit (GE Healthcare Bio-Sciences) was used for cDNA-synthesis with the pd(N)_6_ random hexamers. PCR amplifications were done over intron/exon borders with the ex6pf_m13, ex6pr primers, and a 5′ biotinylated M13 primer (Table S2). The relative expression of the W*W and W*Y transcripts was scored by analyzing the SNP at position chr24:6,268,434 by pyrosequencing. Primer ex6p_seq was used as the sequencing primer and all steps were performed according to manufacturer's protocol (Biotage AB). All samples except skin sample 3 were analyzed in triplicates.

### QTL Analysis

QTL analysis was performed using a red junglefowl×White Leghorn intercross on a series of traits including, growth, egg production, skeletal traits, and behavioral traits. Full descriptions of traits are given elsewhere [18],[30],[31]. Single marker analysis was performed using a fully informative SNP at the *BCDO2* locus (chr24:6,273,428). A general linear model was used to test for significant genotypic differences, with the fixed factors of batch and sex being included for all traits, whilst in the case of morphological traits, body mass was also included as a covariate. Multiple testing correction due to multiple marker intervals was not needed, due to only one marker being tested, though multiple testing of many traits remains an issue.

### Phylogenetic Analysis

Neighbor-Joining trees were constructed from a total of seven independent loci, including *BCDO2*, from sequences aligned by eye using Se-Al [32]. In the case of the control region, an additional phylogenetic analysis was performed using MrBayes 3 [33]. Parameter estimates (including posterior probabilities) and consensus trees resulting from several independent MrBayes runs of at least 10 million generations each were recorded and contrasted. The posterior probabilities listed on the tree in Figure S1 represent the lowest recorded values amongst all the runs. The MrBayes analysis was run firstly using Japanese Quail (AP003195) as an outgroup, and then without an outgroup. The resulting topologies of the trees were identical.

Previously published sequence data [7] for five nuclear markers (four distinct CR1 repeat regions and *OTC* intron 9) were harvested from GenBank (Figures S2, S3, S4, S5, and S6). The analysis of the mitochondrial control region was performed using 20 sequences generated as part of this study combined with 61 previously published sequences representative of the variation found in modern domestic stocks and in the four species of *Gallus*. Lastly, the analysis of the 23.8 kb region encompassing the *BCDO2* gene consisted entirely of newly generated sequences and the publicly available genome sequence. All samples that carried the W*Y allele possessed a 598 bp insertion absent in the published RJF sequence (UCDI) and in most of our red junglefowl sequences. The samples GGA15 (*Gallus gallus jaboiuellei*) and GJF (green junglefowl) carried this fragment located at nucleotide position 6,283,696 on chromosome 24 suggesting that it represent a deletion that happened in the red junglefowl lineage. The sequences OS and L13 were identical. Partial resequencing of the 23.8 kb region was also conducted using a divergent set of domestic breeds homozygous for W*Y which revealed SNPs in complete linkage disequilibrium (data not shown).

A comparison of the topologies derived from the control region of the mitochondrial genome and from the nuclear markers not on chromosome 24, revealed that while Ceylon and grey junglefowl always clustered together, sequences derived from green junglefowl sometimes clustered with Ceylon and grey junglefowl, and sometimes clustered with red junglefowl. This inconsistency is probably the result of the relatively few number of basepairs used in the alignments of each of the markers.

Seven of the nine grey junglefowls used in this study (three from GenBank and six novel sequences) possessed an identical mtDNA control region haplotype that matched one of the most common and globally distributed domestic haplotypes (E1) as identified by Liu *et al.* [3], and a single other grey junglefowl sequence differed by only a single base pair (Figure S1). This evidence suggests that many, if not most of grey junglefowl populations in zoos and in captivity outside of India are descended from ancestors who were mated with domestic hens. Only a single grey junglefowl sequence obtained from a Delhi National Park specimen possessed an mtDNA sequence that was phylogenetically distinct from red junglefowl sequences; the sequence of this bird was replicated in the Uppsala lab. This sample is more likely to be representative of the grey junglefowl not only because of its position on the phylogenetic tree (more closely related to the Ceylon junglefowl sequences), but also because it possesses a 62 bp insert within the control region, a trait shared only by Ceylon junglefowl and not found in any domestic chicken or other species of *Gallus* sequenced thus far. 23.8 kb of the *BCDO2* region was generated from this sample which was found to be heterozygous, one allele of which most closely matched GryJF_04-7 and another which more closely matched TY_216372. The differing nucleotide positions across this region are shown in Figure S7.

Nishibori *et al.* [7] [7] concatenated all the CR1 sequences into one alignment and presented a single tree. Because these sequences rested on different locations within the chicken genome (CR1a, b, c, d, and e on Chr. 24, 1, 1, 5, and 1, respectively), each CR1 sequence was used here to produce a single tree (see Figures S2, S3, S4, S5, and S6) in order to identify possible regions of introgression into the *G. gallus* genome by other species of the genus *Gallus*.

### URL

Information on the chicken genome sequence is available at http://www.genome.ucsc.edu.

### Accession Numbers

The sequence data presented in this paper have been submitted to GenBank with the following accession numbers EU329393–EU329413 and EU334146–334166.

## Supporting Information

Figure S1 A consensus Bayesian tree rooted with a Japanese Quail (not included), depicting the relationships between sequences derived from 725 bp of the mitochondrial control region (the total alignment consists of numerous indels including a 62 base pair insert found in G. sonnerati and G. lafayetii) and posterior probabilities for the major clades. Codes after the GenBank accession numbers refer to the named haplotypes as defined by Liu et al. [Liu YP et al. (2006) Multiple maternal origins of chickens: out of the Asian jungles. Mol Phylogenet Evol 38: 12–19]. The topology of this tree generally matches those derived from the CR1 loci (with the exception of CR1a) and intron 9 of the OTC gene. The fact that only one of the grey junglefowl samples does not possess either the common domestic chicken haplotype E1 or fall into the general red junglefowl/domestic chicken clade suggests that samples of grey junglefowl from zoo collections are unlikely to be pure; the AP006741_G_sonnerati sequence was derived from the Grey junglefowl (Delhi) included in the sequencing of the BCDO2 region (Figure 3).
(0.93 MB TIF)

Figure S2 A neighbor-joining tree depicting the relationships between sequences derived from the CR1b locus on chromosome 1:108725597–108726196. The topology of this tree matches those derived from the other CR1 loci (with the exception of CR1a) and the mtDNA control region sequences.
(0.05 MB TIF)

Figure S3 A neighbor-joining tree depicting the relationships between sequences derived from the CR1c locus located on chromosome 1:186932225–186932682. The topology of this tree generally matches those derived from the other CR1 loci (with the exception of CR1a) and the mtDNA control region sequences.
(0.04 MB TIF)

Figure S4 A neighbor-joining tree depicting the relationships between sequences derived from the CR1d locus located on chromosome 5:14882586–14883036. The topology of this tree generally matches those derived from the other CR1 loci (with the exception of CR1a) and the mtDNA control region sequences.
(0.04 MB TIF)

Figure S5 A neighbor-joining tree depicting the relationships between sequences derived from intron 9 of OTC located on chromosome 1:116461521–116463718. The topology of this tree generally matches those derived from the CR1 loci (with the exception of CR1a) and the control region sequences. The position of an allele belonging to the grey junglefowl from Laos falls inside sequences derived from domestic chickens on both this tree and on the tree derived from the mtDNA control region sequences indicating that this sample is not a pure grey junglefowl.
(0.05 MB TIF)

Figure S6 A neighbor-joining tree depicting the relationships between sequences derived from the CR1a locus located on chromosome 24:5605060–5605972. The contrast between the topology depicted here and the topologies derived from CR1 loci found on other chromosomes is not surprising given that the BCDO2 locus is also found on chromosome 24.
(0.04 MB TIF)

Figure S7 A list of variable positions within the 23.8 kb locus containing the BCDO2 gene between two domestic chickens possessing the yellow skin alleles (WL_AGDA (same as L13) and TY_216372), one grey junglefowl (GryJF_04-7), a Ceylon junglefowl (CJF_2), and the pure grey junglefowl that is heterozygous at this locus (GryJF(Delhi)). The cells in yellow depict the locations where one of the bases identified in the GryJF(Delhi) is also found in any of the other four samples. Cells with no color are locations where the grey junglefowl is the only sample to possess that base at that position. Numbers above the variable positions represent approximate locations starting from the 5′ region along the identified 23.8 kb region.
(0.03 MB TIF)

Table S1 List of bird samples.
(0.28 MB DOC)

Table S2 Primers for SNP analysis, resequencing, and mtDNA analysis.
(0.08 MB DOC)

Table S3 Results of QTL analysis at the BCDO2 locus in an intercross between White Leghorn (W*Y/W*Y) and red junglefowl (W*W/W*W). Only traits reaching statistical significance are presented; no corrections for multiple testing have been performed.
(0.04 MB DOC)
