# Supplementary figures and images for: Identification of the Yellow Skin Gene Reveals a Hybrid Origin of the Domestic Chicken
Source: PLoS Genet. 2008 Feb 29;4(2):e1000010. doi: 10.1371/journal.pgen.1000010 (PMC2265484; doi:10.1371/journal.pgen.1000010)

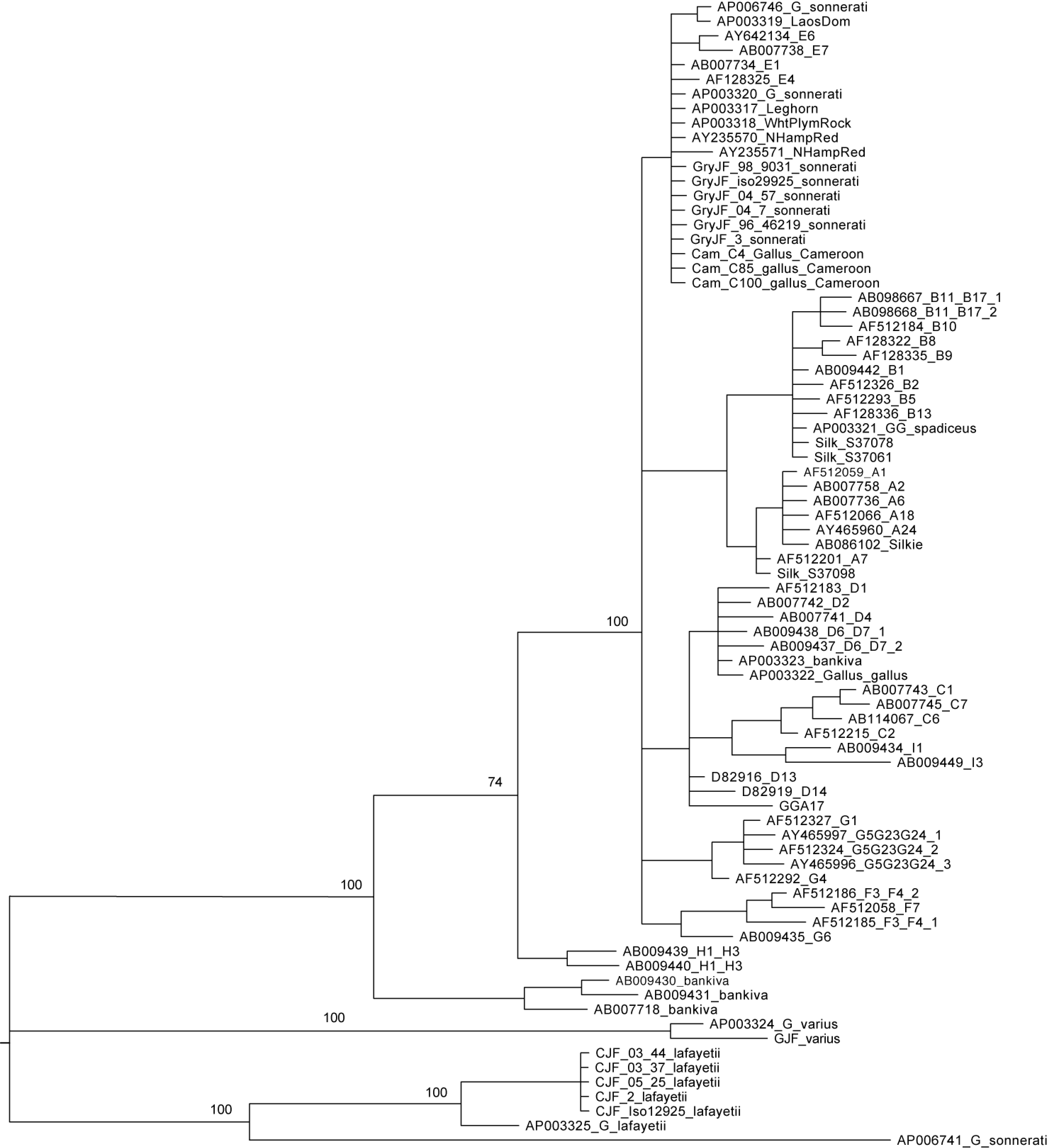

Supplement: Figure S1 — A consensus Bayesian tree rooted with a Japanese Quail (not included), depicting the relationships between sequences derived from 725 bp of the mitochondrial control region (the total alignment consists of numerous indels including a 62 base pair insert found in G. sonnerati and G. lafayetii) and posterior probabilities for the major clades. Codes after the GenBank accession numbers refer to the named haplotypes as defined by Liu et al. [Liu YP et al. (2006) Multiple maternal origins of chickens: out of the Asian jungles. Mol Phylogenet Evol 38: 12–19]. The topology of this tree generally matches those derived from the CR1 loci (with the exception of CR1a) and intron 9 of the OTC gene. The fact that only one of the grey junglefowl samples does not possess either the common domestic chicken haplotype E1 or fall into the general red junglefowl/domestic chicken clade suggests that samples of grey junglefowl from zoo collections are unlikely to be pure; the AP006741_G_sonnerati sequence was derived from the Grey junglefowl (Delhi) included in the sequencing of the BCDO2 region (Figure 3). (0.93 MB TIF) [file pgen.1000010.s001.tif]

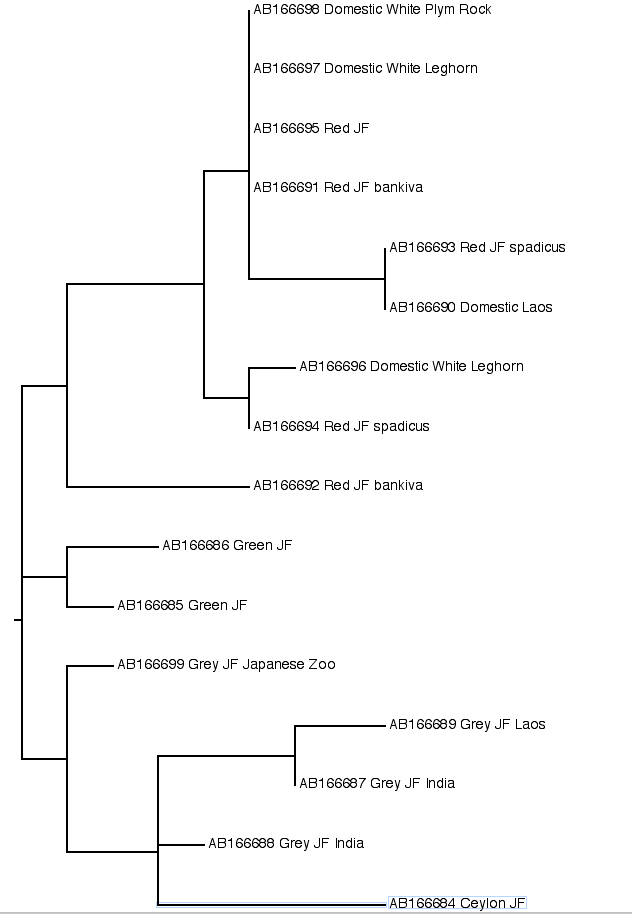

Supplement: Figure S2 — A neighbor-joining tree depicting the relationships between sequences derived from the CR1b locus on chromosome 1:108725597–108726196. The topology of this tree matches those derived from the other CR1 loci (with the exception of CR1a) and the mtDNA control region sequences. (0.05 MB TIF) [file pgen.1000010.s002.tif]

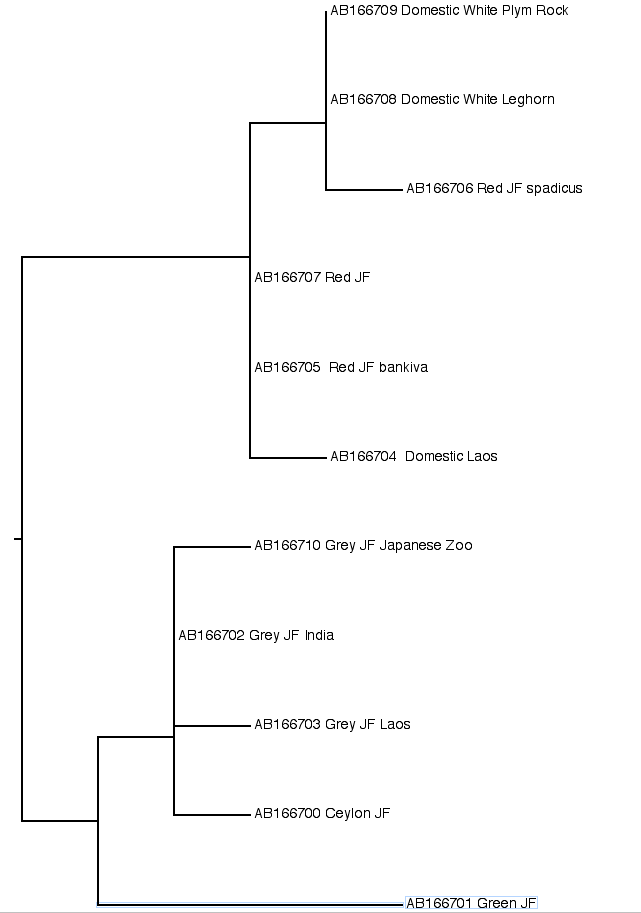

Supplement: Figure S3 — A neighbor-joining tree depicting the relationships between sequences derived from the CR1c locus located on chromosome 1:186932225–186932682. The topology of this tree generally matches those derived from the other CR1 loci (with the exception of CR1a) and the mtDNA control region sequences. (0.04 MB TIF) [file pgen.1000010.s003.tif]

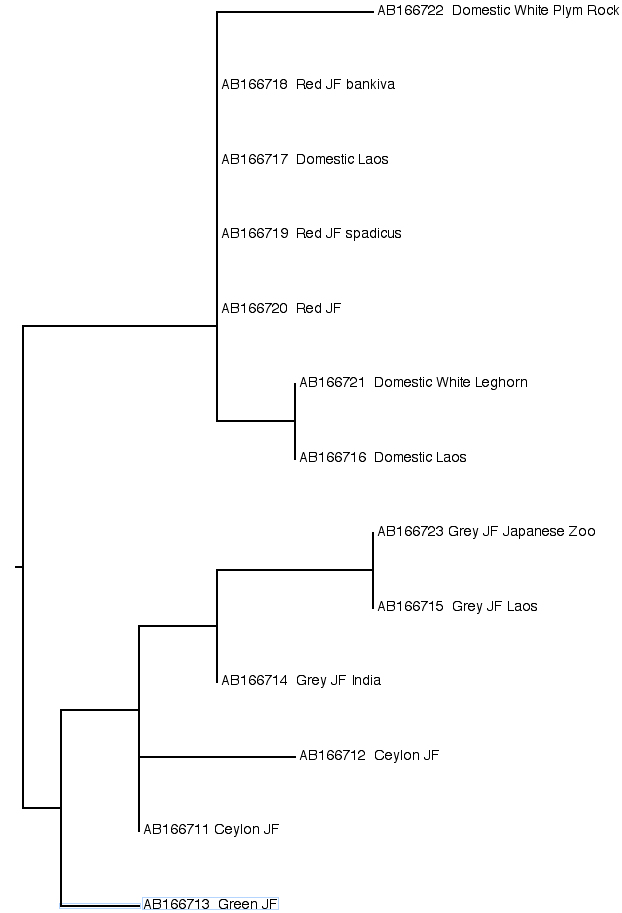

Supplement: Figure S4 — A neighbor-joining tree depicting the relationships between sequences derived from the CR1d locus located on chromosome 5:14882586–14883036. The topology of this tree generally matches those derived from the other CR1 loci (with the exception of CR1a) and the mtDNA control region sequences. (0.04 MB TIF) [file pgen.1000010.s004.tif]

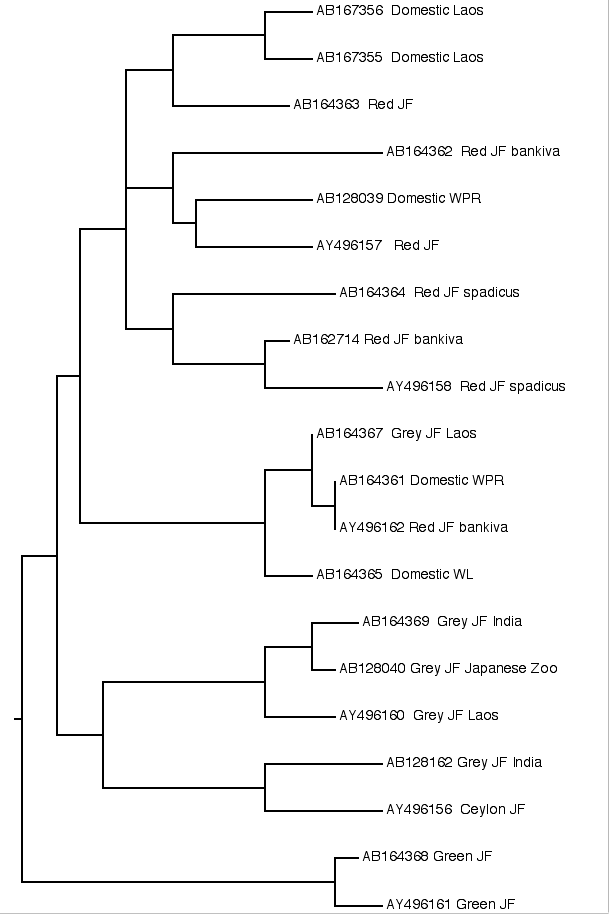

Supplement: Figure S5 — A neighbor-joining tree depicting the relationships between sequences derived from intron 9 of OTC located on chromosome 1:116461521–116463718. The topology of this tree generally matches those derived from the CR1 loci (with the exception of CR1a) and the control region sequences. The position of an allele belonging to the grey junglefowl from Laos falls inside sequences derived from domestic chickens on both this tree and on the tree derived from the mtDNA control region sequences indicating that this sample is not a pure grey junglefowl. (0.05 MB TIF) [file pgen.1000010.s005.tif]

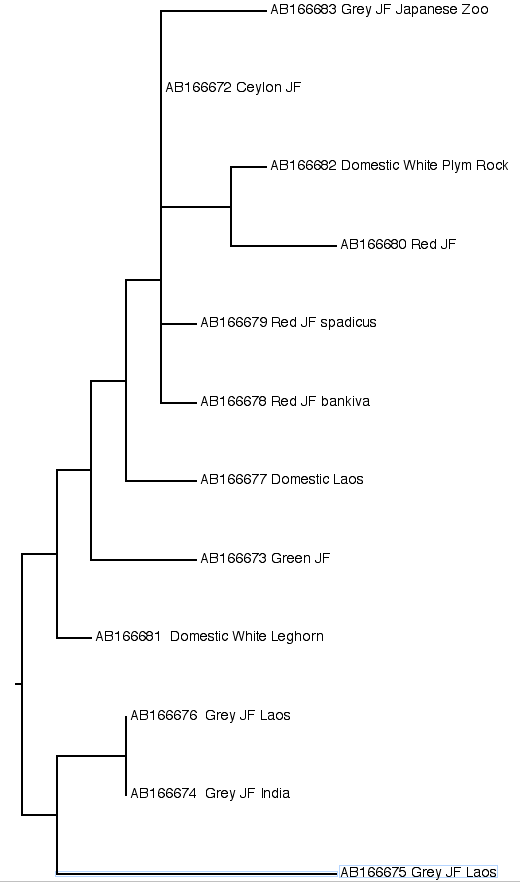

Supplement: Figure S6 — A neighbor-joining tree depicting the relationships between sequences derived from the CR1a locus located on chromosome 24:5605060–5605972. The contrast between the topology depicted here and the topologies derived from CR1 loci found on other chromosomes is not surprising given that the BCDO2 locus is also found on chromosome 24. (0.04 MB TIF) [file pgen.1000010.s006.tif]

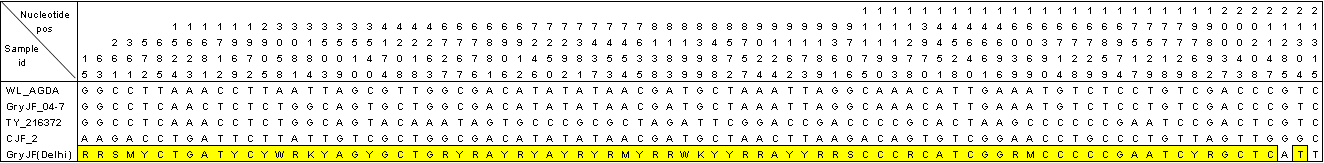

Supplement: Figure S7 — A list of variable positions within the 23.8 kb locus containing the BCDO2 gene between two domestic chickens possessing the yellow skin alleles (WL_AGDA (same as L13) and TY_216372), one grey junglefowl (GryJF_04-7), a Ceylon junglefowl (CJF_2), and the pure grey junglefowl that is heterozygous at this locus (GryJF(Delhi)). The cells in yellow depict the locations where one of the bases identified in the GryJF(Delhi) is also found in any of the other four samples. Cells with no color are locations where the grey junglefowl is the only sample to possess that base at that position. Numbers above the variable positions represent approximate locations starting from the 5′ region along the identified 23.8 kb region. (0.03 MB TIF) [file pgen.1000010.s007.tif]
