# Supplementary material for: Identification of the Yellow Skin Gene Reveals a Hybrid Origin of the Domestic Chicken
Source: PLoS Genet. 2008 Feb 29;4(2):e1000010. doi: 10.1371/journal.pgen.1000010 (PMC2265484; doi:10.1371/journal.pgen.1000010)
Supplement: Table S1 — List of bird samples. (0.28 MB DOC) [file pgen.1000010.s008.doc]

**Supplementary Table 1**. List of bird samples

| **Sample id.** | **Species** | **Subspecies** | **Breed/common name** | **Provider** | **SNP analysis** | **23.8kb region** | **D-loop** |
| --- | --- | --- | --- | --- | --- | --- | --- |
| GGA3 | *G. gallus* | *jaboiuellei* | Red junglefowl | Clères Zoological Park, France | x |  |  |
| GGA12 | *G. gallus* | *jaboiuellei* | Red junglefowl | P. Hermans | x |  |  |
| GGA15 | *G. gallus* | *jaboiuellei* | Red junglefowl | P. Hermans | x | x |  |
| GGA16 | *G. gallus* | *jaboiuellei* | Red junglefowl | P. Hermans | x |  |  |
| GGA17 | *G. gallus* | *jaboiuellei* | Red junglefowl | P. Hermans | x |  | x |
| GGA18 | *G. gallus* | *bankiva?* | Red junglefowl | P. Hermans | x | x |  |
| GGA19 | *G. gallus* | *jaboiuellei* | Red junglefowl | L. Pinceel | x |  |  |
| GGA20 | *G. gallus* | *jaboiuellei* | Red junglefowl | L. Pinceel | x |  |  |
| GGA21 | *G. gallus* | *jaboiuellei* | Red junglefowl | L. Pinceel | x |  |  |
| GGA23 | *G. gallus* | *jaboiuellei* | Red junglefowl | L. Pinceel | x |  |  |
| GGA25 | *G. gallus* | *gallus* | Red junglefowl | L. Pinceel | x |  |  |
| GGA26_G10 | *G. gallus* | *gallus* | Red junglefowl | SLU/P. Jensen | x |  |  |
| GGA27_G11 | *G. gallus* | *gallus* | Red junglefowl | SLU/P. Jensen | x |  |  |
| GGA28_G12 | *G. gallus* | *gallus* | Red junglefowl | SLU/P. Jensen | x |  |  |
| GGA29_G13 | *G. gallus* | *gallus* | Red junglefowl | SLU/P. Jensen | x |  |  |
| GGA30_F7 | *G. gallus* | *gallus* | Red junglefowl | Frösö Zoo, Sweden | x |  |  |
| GGA31_E1.1 | *G. gallus* | *gallus* | Red junglefowl | Ebeltoft zoo, Denmark | x |  |  |
| GGA32_E6 | *G. gallus* | *gallus* | Red junglefowl | Ebeltoft zoo, Denmark | x |  |  |
| GGA33_E7 | *G. gallus* | *gallus* | Red junglefowl | Ebeltoft zoo, Denmark | x |  |  |
| GGA34_E8 | *G. gallus* | *gallus* | Red junglefowl | Ebeltoft zoo, Denmark | x |  |  |
| GGA35_E9 | *G. gallus* | *gallus* | Red junglefowl | Ebeltoft zoo, Denmark | x |  |  |
| GGA36_99-29379 | *G. gallus* | *gallus* | Red junglefowl | Clères Zoological Park, France | x |  |  |
| GGA37_99-29325 | *G. gallus* | *gallus* | Red junglefowl | Clères Zoological Park, France | x |  |  |
| GGA38_G5 | *G. gallus* | *gallus* | Red junglefowl | SLU/P. Jensen |  | x |  |
| GGA38_99-29400 | *G. gallus* | *gallus* | Red junglefowl | Clères Zoological Park, France | x |  |  |
| CJF_03-44 | *G. lafayetii* |  | Ceylon junglefowl | Clères Zoological Park, France |  |  | x |
| CJF_03-37 | *G. lafayetii* |  | Ceylon junglefowl | Clères Zoological Park, France |  |  | x |
| CJF_05-25 | *G. lafayetii* |  | Ceylon junglefowl | Clères Zoological Park, France |  |  | x |
| CJF_2 | *G. lafayetii* |  | Ceylon junglefowl | M. Witterboer, Holland |  | x | x |
| CJF_iso12925 | *G. lafayetii* |  | Ceylon junglefowl | Clères Zoological Park, France |  |  | x |
| GryJF_98-9031^2^ | *G. sonneratii* |  | Grey junglefowl | Clères Zoological Park, France |  |  | x |
| GryJF_96-46219^2^ | *G. sonneratii* |  | Grey junglefowl | Clères Zoological Park, France |  |  | x |
| GryJF_iso29925^2^ | *G. sonneratii* |  | Grey junglefowl | Clères Zoological Park, France |  |  | x |
| GryJF_04-57^2^ | *G. sonneratii* |  | Grey junglefowl | Clères Zoological Park, France |  |  | x |
| GryJF_S1^2^ | *G. sonneratii* |  | Grey junglefowl | M. Witterboer, Holland |  |  |  |
| GryJF_S3^2^ | *G. sonneratii* |  | Grey junglefowl | M. Witterboer, Holland |  |  |  |
| GryJF_04-7 | *G. sonneratii* |  | Grey junglefowl | Clères Zoological Park, France |  | x | x |
| GryJF(Delhi) | *G. sonneratii* |  | Grey junglefowl | Dr A. Fumihito/M. Nishibori, Japan^1^ |  | x | x |
| GJF | *G. varius* |  | Green junglefowl | M. Witterboer, Holland |  | x | x |
| OS652 | *G. gallus* | *domesticus* | OS-line^3^ | S. Kerje | x |  |  |
| OS655/OS | *G. gallus* | *domesticus* | OS-line^3^ | S. Kerje |  | x |  |
| OS658 | *G. gallus* | *domesticus* | OS-line^3^ | S. Kerje | x |  |  |
| OS664 | *G. gallus* | *domesticus* | OS-line^3^ | S. Kerje | x |  |  |
| OS665 | *G. gallus* | *domesticus* | OS-line^3^ | S. Kerje | x |  |  |
| OS667 | *G. gallus* | *domesticus* | OS-line^3^ | S. Kerje | x |  |  |
| L13 | *G. gallus* | *domesticus* | White Leghorn Line 13^4^ | SLU/P. Jensen | x | x | x |
| WL781 | *G. gallus* | *domesticus* | White Leghorn Line 13^4^ | SLU/P. Jensen | x |  |  |
| WL701 | *G. gallus* | *domesticus* | White Leghorn Line 13^4^ | SLU/P. Jensen | x |  |  |
| WL707 | *G. gallus* | *domesticus* | White Leghorn Line 13^4^ | SLU/P. Jensen | x |  |  |
| WL769 | *G. gallus* | *domesticus* | White Leghorn Line 13^4^ | SLU/P. Jensen | x |  |  |
| WEL_3701012 | *G. gallus* | *domesticus* | White-egg layer A | AvianDiv project^5^ | x |  |  |
| WEL_3701082 | *G. gallus* | *domesticus* | White-egg layer A | AvianDiv project^5^ | x |  |  |
| WEL_3701152 | *G. gallus* | *domesticus* | White-egg layer A | AvianDiv project^5^ | x |  |  |
| WEL_3701242 | *G. gallus* | *domesticus* | White-egg layer A | AvianDiv project^5^ | x |  |  |
| WEL_3701291 | *G. gallus* | *domesticus* | White-egg layer A | AvianDiv project^5^ | x |  |  |
| WEL_3701331 | *G. gallus* | *domesticus* | White-egg layer A | AvianDiv project^5^ | x |  |  |
| WEL_3701411 | *G. gallus* | *domesticus* | White-egg layer A | AvianDiv project^5^ | x |  |  |
| WEL_3701501 | *G. gallus* | *domesticus* | White-egg layer A | AvianDiv project^5^ | x |  |  |
| BEL_4 | *G. gallus* | *domesticus* | Brown egg layer B | AvianDiv project^5^ | x |  |  |
| BEL_7 | *G. gallus* | *domesticus* | Brown egg layer B | AvianDiv project^5^ | x |  |  |
| BEL_13 | *G. gallus* | *domesticus* | Brown egg layer B | AvianDiv project^5^ | x |  |  |
| BEL_19 | *G. gallus* | *domesticus* | Brown egg layer B | AvianDiv project^5^ | x |  |  |
| BEL_27 | *G. gallus* | *domesticus* | Brown egg layer B | AvianDiv project^5^ | x |  |  |
| BEL_36 | *G. gallus* | *domesticus* | Brown egg layer B | AvianDiv project^5^ | x |  |  |
| BEL_44 | *G. gallus* | *domesticus* | Brown egg layer B | AvianDiv project^5^ | x |  |  |
| BEL_2 | *G. gallus* | *domesticus* | Brown egg layer D | AvianDiv project^5^ | x |  |  |
| BEL_5 | *G. gallus* | *domesticus* | Brown egg layer D | AvianDiv project^5^ | x |  |  |
| BEL_14 | *G. gallus* | *domesticus* | Brown egg layer D | AvianDiv project^5^ | x |  |  |
| BEL_30 | *G. gallus* | *domesticus* | Brown egg layer D | AvianDiv project^5^ | x |  |  |
| BEL_31 | *G. gallus* | *domesticus* | Brown egg layer D | AvianDiv project^5^ | x |  |  |
| BEL_43 | *G. gallus* | *domesticus* | Brown egg layer D | AvianDiv project^5^ | x |  |  |
| BEL_49 | *G. gallus* | *domesticus* | Brown egg layer D | AvianDiv project^5^ | x |  |  |
| BSL_4 | *G. gallus* | *domesticus* | Broiler sire line D | AvianDiv project^5^ | x |  |  |
| BSL_8 | *G. gallus* | *domesticus* | Broiler sire line D | AvianDiv project^5^ | x |  |  |
| BSL_18 | *G. gallus* | *domesticus* | Broiler sire line D | AvianDiv project^5^ | x |  |  |
| BSL_22 | *G. gallus* | *domesticus* | Broiler sire line D | AvianDiv project^5^ | x |  |  |
| BSL_3 | *G. gallus* | *domesticus* | Broiler sire line D | AvianDiv project^5^ | x |  |  |
| BSL_11 | *G. gallus* | *domesticus* | Broiler sire line D | AvianDiv project^5^ | x |  |  |
| BSL_16 | *G. gallus* | *domesticus* | Broiler sire line D | AvianDiv project^5^ | x |  |  |
| BSL_21 | *G. gallus* | *domesticus* | Broiler sire line D | AvianDiv project^5^ | x |  |  |
| BDL_5 | *G. gallus* | *domesticus* | Broiler dam line D | AvianDiv project^5^ | x |  |  |
| BDL_9 | *G. gallus* | *domesticus* | Broiler dam line D | AvianDiv project^5^ | x |  |  |
| BDL_15 | *G. gallus* | *domesticus* | Broiler dam line D | AvianDiv project^5^ | x |  |  |
| BDL_19 | *G. gallus* | *domesticus* | Broiler dam line D | AvianDiv project^5^ | x |  |  |
| BDL_27 | *G. gallus* | *domesticus* | Broiler dam line D | AvianDiv project^5^ | x |  |  |
| BDL_31 | *G. gallus* | *domesticus* | Broiler dam line D | AvianDiv project^5^ | x |  |  |
| BDL_46 | *G. gallus* | *domesticus* | Broiler dam line D | AvianDiv project^5^ | x |  |  |
| GN7 | *G. gallus* | *domesticus* | Godollo Nhx | AvianDiv project^5^ | x |  |  |
| GN13 | *G. gallus* | *domesticus* | Godollo Nhx | AvianDiv project^5^ | x |  |  |
| GN22 | *G. gallus* | *domesticus* | Godollo Nhx | AvianDiv project^5^ | x |  |  |
| GN24 | *G. gallus* | *domesticus* | Godollo Nhx | AvianDiv project^5^ | x |  |  |
| GN34 | *G. gallus* | *domesticus* | Godollo Nhx | AvianDiv project^5^ | x |  |  |
| GN41 | *G. gallus* | *domesticus* | Godollo Nhx | AvianDiv project^5^ | x |  |  |
| GN45 | *G. gallus* | *domesticus* | Godollo Nhx | AvianDiv project^5^ | x |  |  |
| GN46 | *G. gallus* | *domesticus* | Godollo Nhx | AvianDiv project^5^ | x |  |  |
| Pad_P1 | *G. gallus* | *domesticus* | Padova | AvianDiv project^5^ | x |  |  |
| Pad_P16 | *G. gallus* | *domesticus* | Padova | AvianDiv project^5^ | x |  |  |
| Pad_P36 | *G. gallus* | *domesticus* | Padova | AvianDiv project^5^ | x |  |  |
| Pad_P47 | *G. gallus* | *domesticus* | Padova | AvianDiv project^5^ | x |  |  |
| FF_1F | *G. gallus* | *domesticus* | Friesian Fowl | AvianDiv project^5^ | x |  |  |
| FF_5F | *G. gallus* | *domesticus* | Friesian Fowl | AvianDiv project^5^ | x |  |  |
| FF_13F | *G. gallus* | *domesticus* | Friesian Fowl | AvianDiv project^5^ | x |  |  |
| FF_13M | *G. gallus* | *domesticus* | Friesian Fowl | AvianDiv project^5^ | x |  |  |
| FF_2M | *G. gallus* | *domesticus* | Friesian Fowl | AvianDiv project^5^ | x | x |  |
| FF_9M | *G. gallus* | *domesticus* | Friesian Fowl | AvianDiv project^5^ | x |  |  |
| O_2801051 | *G. gallus* | *domesticus* | Orlov | AvianDiv project^5^ | x |  |  |
| O_2801041 | *G. gallus* | *domesticus* | Orlov | AvianDiv project^5^ | x |  |  |
| O_2801031 | *G. gallus* | *domesticus* | Orlov | AvianDiv project^5^ | x |  |  |
| O_2801141 | *G. gallus* | *domesticus* | Orlov | AvianDiv project^5^ | x |  |  |
| O_2801061 | *G. gallus* | *domesticus* | Orlov | AvianDiv project^5^ | x |  |  |
| O_2801071 | *G. gallus* | *domesticus* | Orlov | AvianDiv project^5^ | x |  |  |
| O_2801081 | *G. gallus* | *domesticus* | Orlov | AvianDiv project^5^ | x |  |  |
| O_2801091 | *G. gallus* | *domesticus* | Orlov | AvianDiv project^5^ | x |  |  |
| O_2801101 | *G. gallus* | *domesticus* | Orlov | AvianDiv project^5^ | x |  |  |
| O_2801111 | *G. gallus* | *domesticus* | Orlov | AvianDiv project^5^ | x |  |  |
| O_2801121 | *G. gallus* | *domesticus* | Orlov | AvianDiv project^5^ | x |  |  |
| O_2801131 | *G. gallus* | *domesticus* | Orlov | AvianDiv project^5^ | x |  |  |
| Silk_S37098 | *G. gallus* | *domesticus* | Silkie | Prof Chen C-F, Prof Lee Y-P. Taiwan |  |  | x |
| Silk_S37061 | *G. gallus* | *domesticus* | Silkie | Prof Chen C-F, Prof Lee Y-P. Taiwan |  |  | x |
| Silk_S37078 | *G. gallus* | *domesticus* | Silkie | Prof Chen C-F, Prof Lee Y-P. Taiwan |  |  | x |
| Low1728 | *G. gallus* | *domesticus* | Low-line/White Plymouth Rock | P. Siegel | x |  |  |
| Low1678 | *G. gallus* | *domesticus* | Low-line/White Plymouth Rock | P. Siegel | x |  |  |
| Low1680 | *G. gallus* | *domesticus* | Low-line/White Plymouth Rock | P. Siegel | x |  |  |
| High1937 | *G. gallus* | *domesticus* | High-line/White Plymouth Rock | P. Siegel | x |  |  |
| High1945 | *G. gallus* | *domesticus* | High-line/White Plymouth Rock | P. Siegel | x |  |  |
| High2068 | *G. gallus* | *domesticus* | High-line/White Plymouth Rock | P. Siegel | x |  |  |
| TY_217901^2^ | *G. gallus* | *domesticus* | Shek-ki | Prof Chen C-F, Prof Lee Y-P. Taiwan |  |  |  |
| TY_220972^2^ | *G. gallus* | *domesticus* | Shek-ki | Prof Chen C-F, Prof Lee Y-P. Taiwan |  |  |  |
| TY_202752 | *G. gallus* | *domesticus* | Shek-ki | Prof Chen C-F, Prof Lee Y-P. Taiwan |  | x |  |
| TY_211316^2^ | *G. gallus* | *domesticus* | Shek-ki | Prof Chen C-F, Prof Lee Y-P. Taiwan |  |  |  |
| TY_216372 | *G. gallus* | *domesticus* | Shek-ki | Prof Chen C-F, Prof Lee Y-P. Taiwan |  | x |  |
| Cam_C4 | *G. gallus* | *domesticus* | Village chicken | J.-C. Fotsa, IRAD |  |  | x |
| Cam_C85 | *G. gallus* | *domesticus* | Village chicken | J.-C. Fotsa, IRAD |  |  | x |
| Cam_C100 | *G. gallus* | *domesticus* | Village chicken | J.-C. Fotsa, IRAD |  |  | x |
| WT2P6 | *G. gallus* | *domesticus* | Westfälischer Totleger | AvianDiv project^5^ | x |  |  |
| WT3P6 | *G. gallus* | *domesticus* | Westfälischer Totleger | AvianDiv project^5^ | x |  |  |
| WT5P6 | *G. gallus* | *domesticus* | Westfälischer Totleger | AvianDiv project^5^ | x |  |  |
| H1P12 | *G. gallus* | *domesticus* | Houdan | AvianDiv project^5^ | x |  |  |
| H4P12 | *G. gallus* | *domesticus* | Houdan | AvianDiv project^5^ | x |  |  |
| H6P12 | *G. gallus* | *domesticus* | Houdan | AvianDiv project^5^ | x |  |  |
| H9P12 | *G. gallus* | *domesticus* | Houdan | AvianDiv project^5^ | x |  |  |
| H10P12 | *G. gallus* | *domesticus* | Houdan | AvianDiv project^5^ | x |  |  |
| D1P14 | *G. gallus* | *domesticus* | Dorking | AvianDiv project^5^ | x |  |  |
| D2P14 | *G. gallus* | *domesticus* | Dorking | AvianDiv project^5^ | x |  |  |
| D3P14 | *G. gallus* | *domesticus* | Dorking | AvianDiv project^5^ | x |  |  |
| D4P14 | *G. gallus* | *domesticus* | Dorking | AvianDiv project^5^ | x |  |  |
| RD1P23 | *G. gallus* | *domesticus* | Red Villafranquina | AvianDiv project^5^ | x |  |  |
| RD3P23 | *G. gallus* | *domesticus* | Red Villafranquina | AvianDiv project^5^ | x |  |  |
| RD6P23 | *G. gallus* | *domesticus* | Red Villafranquina | AvianDiv project^5^ | x |  |  |
| RD7P23 | *G. gallus* | *domesticus* | Red Villafranquina | AvianDiv project^5^ | x |  |  |
| RD26P23 | *G. gallus* | *domesticus* | Red Villafranquina | AvianDiv project^5^ | x |  |  |
| CGP1P24 | *G. gallus* | *domesticus* | Czech Golden Pencilled | AvianDiv project^5^ | x |  |  |
| CGP2P24 | *G. gallus* | *domesticus* | Czech Golden Pencilled | AvianDiv project^5^ | x |  |  |
| CGP3P24 | *G. gallus* | *domesticus* | Czech Golden Pencilled | AvianDiv project^5^ | x |  |  |
| CGP4P24 | *G. gallus* | *domesticus* | Czech Golden Pencilled | AvianDiv project^5^ | x |  |  |
| CGP5P24 | *G. gallus* | *domesticus* | Czech Golden Pencilled | AvianDiv project^5^ | x |  |  |
| A1P46 | *G. gallus* | *domesticus* | Australorp | AvianDiv project^5^ | x |  |  |
| A2P46 | *G. gallus* | *domesticus* | Australorp | AvianDiv project^5^ | x |  |  |
| A3P46 | *G. gallus* | *domesticus* | Australorp | AvianDiv project^5^ | x |  |  |
| A4P46 | *G. gallus* | *domesticus* | Australorp | AvianDiv project^5^ | x |  |  |
| A5P46 | *G. gallus* | *domesticus* | Australorp | AvianDiv project^5^ | x |  |  |

^1^The sample originates from New Delhi Zoo, India.

^2^Approximately 3 kb of the *yellow skin* region from each of these samples were resequenced.

^3^Cole RK (1966) Hereditary hypothyroidism in the domestic fowl. Genetics 53: 1021-1033.

^4^Liljedahl L-E, Kolstad N, Sorensen P, Maijala K (1979) Scandinavian selection and cross-breeding experiment with laying hens. 1. Background and general outline. Acta Agric Scand 29: 273-285.

^5^Hillel J, Groenen MAM, Tixier-Boichard M, Korol AB, David L, et al. (2003) Biodiversity of 52 chicken populations assessed by microsatellite typing of DNA pools. Genet Sel Evol 35: 533-557.

In summary, six birds with *yellow skin* were used for sequencing the 23.8 kb region and two of them were domestic. In addition 10 domestic breeds with *yellow skin* were included in the SNP screen. Four chickens (one domestic) with *white skin* were used for sequencing the 23.8 kb region and eight domestic breeds with *white skin* and 23 red junglefowls were included in the SNP screen.
