# Supplementary material for: Identification of the Yellow Skin Gene Reveals a Hybrid Origin of the Domestic Chicken
Source: PLoS Genet. 2008 Feb 29;4(2):e1000010. doi: 10.1371/journal.pgen.1000010 (PMC2265484; doi:10.1371/journal.pgen.1000010)
Supplement: Table S2 — Primers for SNP analysis, resequencing, and mtDNA analysis. (0.08 MB DOC) [file pgen.1000010.s009.doc]

**Supplementary Table 2**. Primers for SNP analysis, resequencing, and mtDNA analysis

| **Genotyping of SNPs by pyrosequencing** |  |  |
| --- | --- | --- |
|  |  |  |
| **Name and SNP position** | **Forward primer sequence** | **Reverse primer sequence/seq primer** |
| intron10_pyro (GGA chr24:6,264,085) | M13-ACTCAAAAGGGTGAACAGTGG | CCAAATCTATCAAGCACAATAACAT |
| BCDO2_pyro( GGA chr24:6,273,428 ) | M13-TCTTACTCGGTGGATCAAAGC | ATTGTGGTCTCAGAATTTGGG |
| 10kbup2_pyro (GGA chr24:6,287,900) | M13-GGGTCCCAGAGTTCTTTCCT | AAGAGCATCTGAGCCATTCC/GGTTCCTTTGAAACTGGAGATGT^1^ |
| M13-Bio | Bio-CACGACGTTGTAAAACGAC |  |
|  |  |  |
| **Genotyping by SingleBaseExtension** |  |  |
|  |  |  |
| **Name** | **Forward primer sequence** | **Reverse primer sequence** |
| OL02-358 | CTTCTGGCAGCACGATGAG | GAGTTCGTCCAGGAAGGAGA |
| OL03_815^1^ | (T)52CTCCTCCGTCCACTTGGC |  |
|  |  |  |
| **Differential expression** |  |  |
|  |  |  |
| **Name** | **Forward primer sequence** | **Reverse primer sequence** |
| ex6pf_m13 | CACGACGTTGTAAAACGACGGCCACTGCTCATCCTCA | TCAGCTTGATGGGCTGCT/TGTTGGAGCAATGGAGCA^1^ |
|  |  |  |
| **PCR and resequencing of mitochondrial DNA** |  |  |
|  |  |  |
| **Name** | **Forward primer sequence** | **Reverse primer sequence** |
| GalCR_L16750 | AGGACTACGGCTTGAAAAGC | CATCTTGGCATCTTCAGTGCC |
| GalCR_intrev1^2^ | GAGTGAGATTTATGTCCTGC |  |
| GalCR_intf1^2^ | CCCATTCTTTCCCCCTAC |  |
| GalCR_intrev2^2^ | GACACGAGAGGACTAGGA |  |
| GalCR_intf2^2^ | ACATACAAACTACCGCATAA |  |
|  |  |  |
| **PCR and resequencing of 23.8 kb** |  |  |
|  |  |  |
| **Name** | **Forward primer sequence** | **Reverse primer sequence** |
| BCDO2_ex11 | AGAAGTTGTGCCCTTGAGT | TGTTTATTTCAGCCCATTT |
| BCDO2_ex10 | GAGAAAGCAAGGACAGATTG | GGATGCAAAACCTTAGAGTG |
| Intron10 | CCTGCCAAATCTATCAAGCA | GGAAACAGAAAGGGTTGCAT |
| BCDO2_ex9 | AACCAATCAGTTTCTTGTGG | TCTGTACACTGGTGAAAGCA |
| intron8_2 | GGAGAGTGGCATTTTGGGTA | AAGGGCAGTTTCTGCAACAT |
| intron8_1 | TGCTTCCAGACAGCTCAGAA | CCCATGCTGGCTGAGTTATT |
| ex8 | GAAGTGCATGTGAGGACCAA | ATGGATGGAGCCATGGATAG |
| BCDO2_ex7 | ATAATTGAGGTCCCTCCACA | AATACATAGGCAGAGCCAGA |
| BCDO2_ex6 | ATGGCAGTTATCAACCAGAT | TTCCCAGTGCGTTTATCTAC |
| BCDO2_4445 | GGGTTCAGCACCACTGAGAT | TTTCCCAGGTCTTCTTCCAA |
| BCDO2_ex5 | GACAACATTCCTGCAGTGTC | AGAAGAAAACATATCCCAGAAA |
| intron4 | GCCCTAATGTGGGATCTGAA | CTTTGTAGCCACTGGGTGGT |
| intron4h^3^ | CATGGTACCAGGAGCTCACA |  |
| BCDO2_6730 | CTGATGCAATGCCTCTCTCA | GGTGGTTTCCGTTTGTCATT |
| BCD02_ex4 | AAGAAGCTACAGAGCACCAG | AGAACAGATGGGAAGACTGA |
| BCDO2_7700 | GCATTGTCACTTGGCTCTGA | TGGCTGGGGAAGACTAGAGA |
| BCDO2_ex3 | GGAAGATGCACATTCACTTT | ATTGTCCAGAGGATGTTCAG |
| BCDO2_B | TTGGGCTAAGAAGTGGGAAG | CATGAGCTCGTCATGGTCAA |
| BCDO2_9470 | CATGCCATCAAACCAGTGAT | AGCAACTGGCCAAAGACAAT |
| BCDO2_A | GGGAAGTCTGGGTGTTTTTAAG | GGGGATTCTGAGTACCATTCTT |
| BCDO2_10685 | GCAAGGTGAACTGCTCTTCC | GGACAGCATATCCTGCACAA |
| BCDO2_11360 | TGCAAACAGCAAGACAGAGC | CTGTGGGCAGAAAATGATGA |
| BCDO2_12120 | AATTCTGGGGAGGGGATGTA | GTCATGCATGTGTGCCTCTC |
| BCDO2_12655 | GATTGCAGGAGCTCAGAAGG | GAAGCATCTGCAGTGGTGAA |
| BCDO2_12245 | TCATCATTTTCTGCCCACAG | ACGACCCTTTTCTGCAGTTG |
| BCDO2_12635 | CTCAAACTCACCGGGACAAT | GAAGCATCTGCAGTGGTGAA |
| BCDO2_13480 | TGCTTGGCATACAGAAGTCC | TGCAGCTCTCCAGGAAGTTT |
| BCDO2_14105 | CCCAACATTAGTGCCCTTAG | GATACTCCCTTGCCTTGCTA |
| BCDO2_EST-ex2 | CCAAGTTTTCCTCCTCCTAT | CATTGCAAAATGCTAATTCA |
| BCDO2con1&2 | CGCAAGTTAGATGAAGATCC | TGTTGTAACAGGGACGAAGT |
| BCDO2_15835 | GCCACCATAGCTGTGTTTAGG | CAATTGGTGACAGGCAGAGA |
| BCDO2con1 | AGCCTGGTATTGTTTCTTCA | TTTCCTAGCCCTTATTCACA |
| BCDO2_17050 | TCCTTGCTAACAGGCAGTGA | CGTTTCCCCTCAGAATTCAA |
| BCDO2_17605 | ACAAAGGAAGGTGCAGGAGA | TAGCGATCAGAAGGGAGGAA |
| BCDO2_EST | CATCATTTCCTCCCACCT | ACCTTAACATGAAGCAGCAG |
| BCDO2_ex1_up | CTGTAGCTGGAGAACGTACC | ACTGGGTAAGGGAGAAAAAG |
| BCDO2_19425 | CTGTCCTTTTTATCACTGATGCT | AATCCAATAAGAAATGGTAGAACTG |
| BCDO2ex1up2 | ATTATTCCCTTTTCCCACTC | AAGCATGTGACAATCTTCC |
| BCDO2_20055 | CCTCAGATGCTTCCACGATT | CGAAGTGCCTACGCATGTAA |
| BCD02_ex1_up3 | AACTGCAGGAAGATTGTCAC | GTGACTGAAAAGAGGAGCTT |
| BCDO2_21500 | GAAAAGCTGAAGGTGCATCA | AGAGGAACCCATCACAGCAA |
| BCDO2_22310 | TTGATCAAACACTGTTGTTACTCTG | AAAGAGCTGGAGGTGAGCAG |
| BCDO2_22995 | TGGCTTGAAACAAGCTCAGA | AGGTGTCCGCTCATGAAAAC |
| BCDO2_10kb_up | TTCGCTTCTGGTTTTAAGAG | ACCAGCAGAGTAAAACCTCA |

All primers are listed 5’-3’.

^1^Primer used for typing of SNP.

^2^Primer used for sequencing of the PCR product generated with GalCR_L16750 forward and reverse (GalCR_rev) primers.

^3^Primer used for sequencing of the PCR product generated with intron4 forward and reverse primers.
