# Supplementary material for: Identification of the Yellow Skin Gene Reveals a Hybrid Origin of the Domestic Chicken
Source: PLoS Genet. 2008 Feb 29;4(2):e1000010. doi: 10.1371/journal.pgen.1000010 (PMC2265484; doi:10.1371/journal.pgen.1000010)
Supplement: Table S3 — Results of QTL analysis at the BCDO2 locus in an intercross between White Leghorn (W*Y/W*Y) and red junglefowl (W*W/W*W). Only traits reaching statistical significance are presented; no corrections for multiple testing have been performed. (0.04 MB DOC) [file pgen.1000010.s010.doc]

**Supplementary Table 3**. Results of QTL analysis at the *BCDO2* locus in an intercross between White Leghorn (*W*Y/W*Y*) and red junglefowl (*W*W/W*W*). Only traits reaching statistical significance are presented; no corrections for multiple testing have been performed.

|  | | | | *BCDO2* genotype (least square means ± SE) | | |
| --- | --- | --- | --- | --- | --- | --- |
| Trait | n | F | P | *W/W* | *W/Y* | *Y/Y* |
| Number of eggs laid per week | 334 | 3.2 | 0.043 | 4.81±0.15 | 5.19±0.08 | 5.17±0.13 |
| CT_cortical_density (mg/mm^3^) | 286 | 5.5 | 0.005 | 1.110±0.004 | 1.124±0.002 | 1.116±0.004 |
| 3-point-bend displacement at failure (mm) | 258 | 5.4 | 0.005 | 6.11±0.27 | 6.69±0.24 | 5.58±0.19 |
| Rope test - number of jumps performed | 712 | 4.1 | 0.017 | 0.95±0.08 | 0.75±0.05 | 0.68±0.06 |

n=number of F_2_ birds; F=F statistics; P=probability value, not corrected for multiple testings.
